# Blood and Brain Biochemistry and Behaviour in NTBC and Dietary Treated Tyrosinemia Type 1 Mice

**DOI:** 10.3390/nu11102486

**Published:** 2019-10-16

**Authors:** Willem G. van Ginkel, Danique van Vliet, Els van der Goot, Martijn H. J. R. Faassen, Arndt Vogel, M. Rebecca Heiner-Fokkema, Eddy. A. van der Zee, Francjan J. van Spronsen

**Affiliations:** 1 Beatrix Children’s Hospital, University Medical Center Groningen, University of Groningen, 9700 RB Groningen, The Netherlands; w.g.van.ginkel@umcg.nl (W.G.v.G.); d01.vliet@umcg.nl (D.v.V.); 2 Molecular Neurobiology, Groningen Institute for Evolutionary Life Sciences, University of Groningen, 9747 AG Groningen, The Netherlands; e.van.der.goot@rug.nl (E.v.d.G.); e.a.van.der.zee@rug.nl (E.A.v.d.Z.); 3 Department of Laboratory Medicine, University Medical Center Groningen, University of Groningen, 9700 RB Groningen, The Netherlands; h.j.r.van.faassen@umcg.nl (M.H.J.R.F.); m.r.heiner@umcg.nl (M.R.H.-F.); 4 Department of Gastroenterology, Hepatology and Endocrinology, Hannover Medical School, 30625 Hannover, Germany; Vogel.Arndt@mh-hannover.de

**Keywords:** tyrosinemia type 1, FAH, mice, large neutral amino acids, brain biochemistry, phenylalanine, tyrosine, NTBC

## Abstract

Tyrosinemia type 1 (TT1) is a rare metabolic disease caused by a defect in the tyrosine degradation pathway. Neurocognitive deficiencies have been described in TT1 patients, that have, among others, been related to changes in plasma large neutral amino acids (LNAA) that could result in changes in brain LNAA and neurotransmitter concentrations. Therefore, this project aimed to investigate plasma and brain LNAA, brain neurotransmitter concentrations and behavior in C57 Bl/6 fumarylacetoacetate hydrolase deficient (FAH−/−) mice treated with 2-(2-nitro-4-trifluoromethylbenoyl)-1,3-cyclohexanedione (NTBC) and/or diet and wild-type mice. Plasma and brain tyrosine concentrations were clearly increased in all NTBC treated animals, even with diet (*p* < 0.001). Plasma and brain phenylalanine concentrations tended to be lower in all FAH−/− mice. Other brain LNAA, were often slightly lower in NTBC treated FAH−/− mice. Brain neurotransmitter concentrations were usually within a normal range, although serotonin was negatively correlated with brain tyrosine concentrations (*p* < 0.001). No clear behavioral differences between the different groups of mice could be found. To conclude, this is the first study measuring plasma and brain biochemistry in FAH−/− mice. Clear changes in plasma and brain LNAA have been shown. Further research should be done to relate the biochemical changes to neurocognitive impairments in TT1 patients.

## 1. Introduction

Tyrosinemia Type 1 (TT1; McKusick 27670) is an inborn error of tyrosine metabolism caused by a deficiency of the enzyme fumarylacetoacetate hydrolase (FAH). Due to this deficiency, toxic products such as fumarylacetoacetate, maleylacetoacetate, succinylacetoacetate and succinylacetone (SA) accumulate proximal to the enzymatic defect, mainly causing acute liver failure, development of hepatocellular carcinoma at a young age, renal tubulopathy and/or porphyria like syndrome. However, the outcome improved tremendously when 2-(2-nitro-4-trifluoromethylbenoyl)-1,3-cyclohexanedione (NTBC) was introduced as a new treatment option in 1992. The herbicide NTBC inhibits the enzyme 4-hydroxyphenylpyruvate dioxygenase (HPPD), an enzyme proximal to the primary enzymatic defect. Thus, NTBC prevents the formation of the toxic products mentioned before, but leads to increased tyrosine concentrations, making dietary restriction of tyrosine and its precursor phenylalanine again necessary [1,2,3].

With this combined treatment, most clinical problems could be prevented in TT1 patients. However, the last few years, neurocognitive and behavioral problems were reported in these patients [4]. So far, the pathophysiological mechanisms underlying these neurocognitive and behavioral problems in TT1 patients are not fully understood, although biochemical differences and treatment with the herbicide NTBC potentially play a central role. The nine large neutral amino acids (LNAA), including tyrosine and phenylalanine, are transported across the blood-brain barrier in a competitive way [5]. In this way, the high blood tyrosine, and low blood phenylalanine concentrations that are often found, could lead to changes in brain LNAA concentrations and consequently to changes of monoaminergic neurotransmitters synthesis [6,7]. This competitive transport of LNAA has been shown to be important in other inborn errors of metabolism such as phenylketonuria (PKU) and maple syrup urine disease (MSUD) [8,9]. Besides, changes in brain LNAA and neurotransmitter concentrations, NTBC and toxic metabolites such as SA have both been associated with cognitive and behavioral deficiencies as well [10,11,12]. Brain LNAA and neurotransmitter concentrations are, however, difficult to examine in TT1 patients. Therefore, to contribute to a better understanding of the pathophysiological mechanisms underlying the cognitive and behavioral problems in TT1 patients, the present project aimed to investigate: (1) Blood and brain biochemistry in FAH−/− mice; (2) the effect of different doses of NTBC and dietary treatment on blood and brain biochemistry; and (3) evaluate behavioral differences between different groups of FAH−/− and wild-type (WT) mice.

## 2. Materials and Methods

### 2.1. Animals

C57 Bl/6 FAH exon 5 knockout mice were kindly provided by Dr. Grompe from the department of Pediatrics of the Oregon Health and Science University, USA and Prof. Dr. Vogel from the department of Gastroenterology of Medical University Hannover, Germany. All mice were originally from the FAH−/− strain previously described [13]. Both groups of mice were crossbred with each other at least two generations before the experiment took place. The FAH−/− mice used for this experiment were obtained by homozygous mating. As FAH−/− mice need NTBC to be administered prenatally, WT C57 Bl/6 mice without NTBC administration were obtained by separate breeding to function as control group. In addition, WT mice receiving NTBC pre- and post-natal were obtained by heterozygous (FAH+/−) mating. All mice were housed in the same room and handled by the same person during breeding.

At three weeks of age, a small piece of the ear was taken to perform genetic analyses by PCR to confirm FAH−/− or WT background. In total, 56 FAH−/− mice (28 male and 28 female) and 28 WT (14 male and 14 female) were included in the study when they were four weeks of age. Mice were housed individually in a room controlled for temperature (21 ± 1 °C) on a 12 h light-dark cycle (7:30 am–7:30 pm). Cages were equipped with nesting material and a paper roll. Food pellets and water (with or without dissolved NTBC) were provided ad libitum. This study was carried out in strict accordance with the recommendations in the Guide for the Care and Use of Laboratory Animals of the National Institutes of Health. The protocol was approved by the Institutional Animal Care and Use Committee of the University of Groningen.

### 2.2. Experimental Design

At four weeks of age, mice were weaned and assigned to one of the following six experimental groups: (1) FAH−/− mice treated with 8 mg/L NTBC; (2) FAH−/− mice treated with 32 mg/L NTBC; (3) FAH−/− mice treated with 8 mg/L NTBC and a diet; (4) FAH−/− mice treated with 32 mg/L NTBC and a diet; (5) WT mice receiving 8 mg/L NTBC without a diet; (6) untreated WT mice receiving water with neither NTBC nor a diet.

Body weight and food intake were measured daily during the first week of the experiment and weekly afterwards. New NTBC water was provided at least once a week. All mice were handled for two minutes for five consecutive days before the start of the behavioral paradigm at 12 weeks of age. The following behavioral tests were performed in chronological order: (1) Open field (OF) to assess exploration and anxiety-like behavior; (2) novel object recognition (NOR) to evaluate learning and memory; (3) elevated plus maze (EPM) as a second measure of anxiety and (4) forced swim test (FST) to assess depressive-like behavior. All mice started with the OF and NOR, followed by the EPM and FST seven and eight days later respectively. All behavioral tests were executed as described by Bruinenberg et al. 2016 [14]. At 15 weeks of age, 11 days after the last behavioral experiment, mice were euthanized by combined heart puncture and decapitation under inhalation-anesthetics with isoflurane 1–3 h after the beginning of the light phase and blood and brain tissue was collected for further analyses.

### 2.3. NTBC

NTBC was provided by Yecuris Inc. A stock solution with a concentration of 2 mg/mL was made and stored refrigerated and protected from light up to two months. To make drinking water, a small amount of the stock solution was added to autoclaved water to reach the appropriate concentration. This solution was refrigerated, light protected and stored maximally a week before new drinking water was made. All pregnant FAH−/− dams were treated with 16 mg/L NTBC through the drinking water. In this way, all FAH−/− pups and WT pups that needed to receive NTBC during the experiment, received NTBC through the mother pre- and post-natal, up to weaning age. After weaning, all mice received autoclaved water with or without NTBC depending on in which experimental group they were.

### 2.4. Experimental Diets

The basic diet for all mice was AIN-93 M which was administered in unadjusted form to the FAH−/− and WT control groups treated without a diet. The dietary treated mice received a diet that was composed in such a way to achieve a reduction of 75% in tyrosine and 50% in phenylalanine. It was produced by a 75% reduction in casein and supplemented by the essential amino acid mixture with the same composition as being used in TT1 patients (without phenylalanine and tyrosine), using a conversion factor of 35% for single amino acids at the expense of cornstarch. Extra phenylalanine was added to the diet to reach a reduction of 50% compared to the control diet. The amino acid supplement was kindly provided by Nutricia and diets were prepared by Research Diet Services B.V. (Wijk Bij Duurstede, the Netherlands).

### 2.5. Biochemical Analyses

Blood was obtained for biochemical analyses by heart puncture. Two small drops of the collected blood were transferred to bloodspot cards, the remaining blood was collected in heparin tubes. Blood was centrifuged at 1800 *g* for 10 min and plasma was collected and stored at –80 °C until analysis. Bloodspot cards were dried for 24 h, at room temperature while protected from light. Afterwards, all bloodspot cards were stored at –80 °C until further analyses were performed.

The cerebrum was snap frozen in liquid nitrogen and stored at −80 °C until further preparation. Frozen cerebrum was first crushed in liquid nitrogen and brain tissue was divided into aliquots. Frozen brain powder for amino acid measurements was processed to 20% (weight: volume (w:v)) homogenates in phosphate-buffered saline (pH 7.4), and for tryptophan, indole and catecholamine measurements to 2% (w:v) homogenates in acetic acid (0.08 M). Brain homogenates were sonicated on ice at 10 W. Next, samples were centrifuged at 12,800 *rpm* for 10 min (4 °C), and the supernatant/internatant was put on ice to be used for further analyses. Plasma and brain amino acid analyses and analyses of monoaminergic neurotransmitters were thereafter done as described by van Vliet et al. 2015 [9]. Bloodspot NTBC and SA concentrations were determined with LC-MS/MS as described by Kienstra et al. 2018 [15]. SA concentrations were divided in quantitatively detectable (≥0.3 µmol/L) and non-quantitatively detectable (<0.3 µmol/L).

### 2.6. Behavioral Analyses

The OF test was analyzed by using Ethovision, measuring the total distance moved (“exploration”) and the time the animal spent in more sheltered zones (“anxiety”). The explorative behavior during the NOR was analyzed using ELINE (an in house developed scoring system). The NOR discrimination index was calculated using the time exploring the new object divided by the total explorative time multiplied by 100. The EPM was divided into three different zones: Center, open arm and closed arm. The number of entries and time spent in each zone was calculated manually using ELINE. Afterwards, a percentage score of the time spent in the different arms was calculated. Regarding the FST, floating time, expressed as an immobile state with only small movements to keep balance, was recorded with ELINE and a percentage score was calculated afterwards.

### 2.7. Statistics

Statistical analyses were performed using IBM SPSS Statistics for Windows, version 23.0. All the tests were performed 2-sided and α < 0.05 was considered significant. Data were expressed as means ± SDs unless otherwise indicated. In case of a non-normal distribution, log-transformed data was used. Differences in body weight at the start of the experiment were analyzed using Welch’s ANOVA. The effect of dietary treatment on body weight during the experiment was analyzed by repeated-measures ANOVA with Tukey’s post hoc analysis, with one between-subject factor (treatment group, 6 levels), one within-subjects factor (time, 12 levels). Plasma and brain biochemistry were analyzed using Welch ANOVA with Games–Howell post hoc tests. In addition, presumed brain influx of each single LNAA was calculated based on LNAA plasma concentrations using the LAT-1 transport characteristics previously described by Smith and by Strauss et al. [16,17]. The presumed brain LNAA influx and plasma LNAA concentrations were correlated to the measured brain LNAA concentrations using linear regression analyses. Results on behavior were compared between the different groups using two-way ANOVA analyses with treatment group and gender as factors or with separate analyses with Welch statistic in case of unequal variances.

## 3. Results

### 3.1. General Health and Dietary Intake

Out of all 84 mice, one mouse (WT mouse receiving NTBC) showed excessive grooming and one mouse (FAH−/− receiving 32 mg/L NTBC without diet) showed signs of malocclusion at the end of the experiment. Both were excluded from analyses. All other mice were healthy based on general behavior and weight gain. Weight at the start of the experiment did not significantly differ between the different experimental groups (*p =* 0.490). In addition, body weight curves during the experiment did not significantly differ between the different groups of mice as well (*p* = 0.702). The experimental diet and normal AIM-93 diet were well tolerated by the mice and the total food intake during the experiment did not differ between the different group of mice (*p =* 0.513).

### 3.2. Plasma Biochemistry

**Figure 1:**
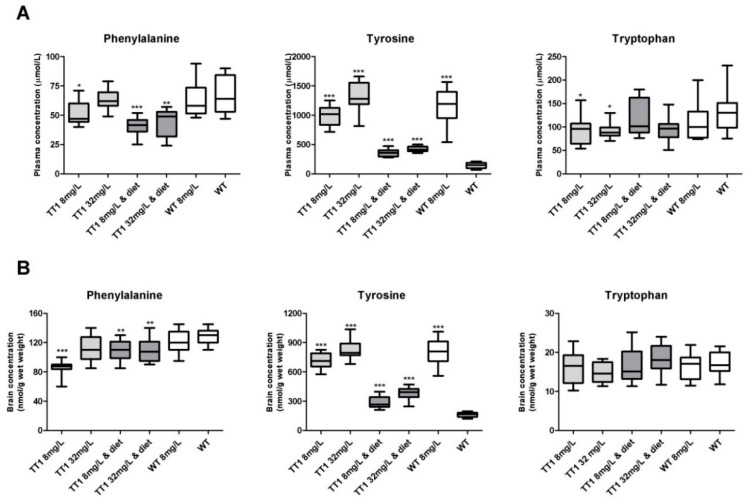
Plasma (**A**) and brain (**B**) phenylalanine, tyrosine and tryptophan concentrations. Untransformed data are expressed as boxplots (min–max whiskers). Numbers of mice are *n* = 13 or *n* = 14 for all treatment groups. For each large neutral amino acids (LNAA), significant differences between the different groups of mice and wild-type (WT) mice without 2-(2-nitro-4-trifluoromethylbenoyl)-1,3-cyclohexanedione (NTBC) are shown only, whereas other significant differences are explained in the main text. * *p* < 0.05, ** *p* < 0.01 and *** *p* < 0.001 (two-sided).

**Figure 2:**
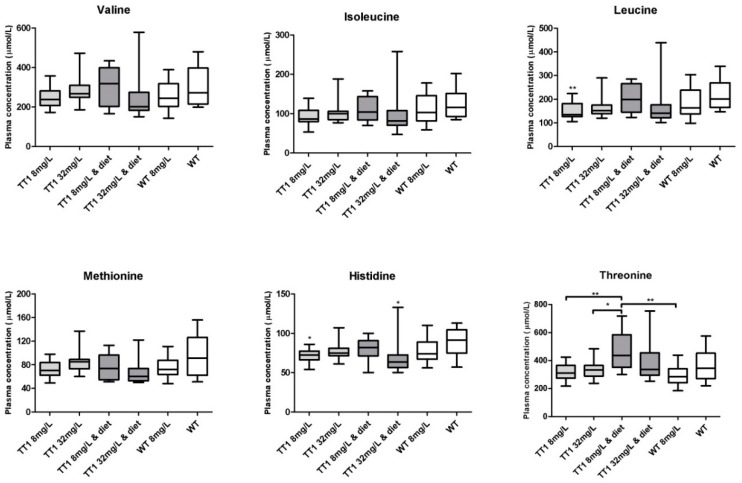
Plasma concentrations of the non-phenylalanine, tyrosine and tryptophan LNAA. Untransformed data are expressed as boxplots (min–max whiskers). Numbers of mice are *n* = 13 or *n* = 14 for all treatment groups. * *p* < 0.05, ** *p* < 0.01 (two-sided).

Figure 1A shows plasma phenylalanine, tyrosine and tryptophan concentrations in WT and FAH−/− mice treated with different doses of NTBC with or without diet. FAH−/− mice treated with 8 mg/L NTBC showed lower plasma phenylalanine (*p* = 0.025), higher tyrosine (*p* < 0.001), and lower tryptophan concentrations (*p* = 0.043), when compared to untreated WT mice. In FAH−/− mice treated with 32 mg/L NTBC, plasma phenylalanine was significantly higher than in FAH−/− mice treated with 8 mg/L NTBC (*p =* 0.019) and not significantly different from concentrations seen in WT mice (*p* = 0.986). In addition, plasma tyrosine concentrations were higher in FAH−/− mice treated with 32 mg/L NTBC than in mice treated with 8 mg/L NTBC (*p* = 0.004), while plasma tryptophan were not different (*p* = 1.000). Comparisons between FAH−/− mice treated with 8 mg/L or 32 mg/L NTBC to the mice receiving the same amount of NTBC and diet, revealed lower plasma phenylalanine concentrations in both groups of FAH−/− mice treated with diet (*p* < 0.05) and lower plasma tyrosine concentrations (*p* < 0.001), while plasma tryptophan concentrations were not different. When WT mice treated with NTBC were compared to untreated WT mice, plasma tyrosine concentrations were higher (*p* < 0.001), while plasma phenylalanine and tryptophan were not different. All other plasma LNAA concentrations and significant differences between the different groups of mice are shown in Figure 2.

Blood NTBC concentrations were significantly lower in all groups of mice treated with 8 mg/L NTBC when compared to all groups of mice treated with 32 mg/L NTBC (*p* < 0.001). SA was quantitatively detectable in 12 out of 14 (86%) FAH−/− mice treated with 8 mg/L alone, while in all other groups of FAH−/− mice, SA was only quantitatively detectable in three or four mice per experimental group (consisting of 13–14 mice (21–29%)).

### 3.3. Brain Biochemistry

**Figure 3:**
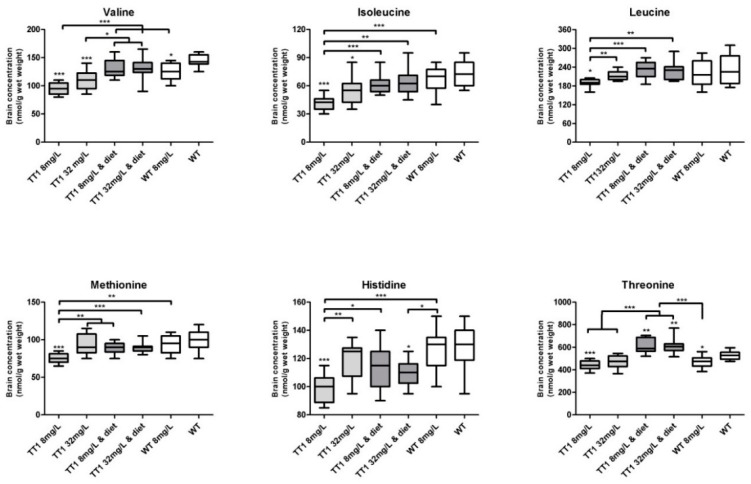
Brain concentrations of the non-phenylalanine, tyrosine and tryptophan LNAA. Untransformed data are expressed as boxplots (min–max whiskers). Numbers of mice are *n* = 13 or *n* = 14 for all treatment groups. * *p* < 0.05, ** *p* < 0.01 and *** *p* < 0.001 (two-sided).

Figure 1B shows brain phenylalanine, tyrosine and tryptophan concentrations in WT and FAH−/− mice treated by different doses of NTBC with or without diet. Compared to untreated WT mice, FAH−/− mice treated with 8 mg/L NTBC showed lower brain phenylalanine (*p* < 0.001) and higher tyrosine (*p* < 0.001) concentrations, while brain tryptophan concentrations were not different. In FAH−/− mice treated with 32 mg/L NTBC, brain phenylalanine concentrations were higher than in FAH mice treated with 8 mg/L NTBC (*p* = 0.001), although brain phenylalanine concentrations still tended to be lower than in untreated WT mice (*p* = 0.057). In addition, brain tyrosine concentrations were higher in FAH mice treated with 32 mg/L NTBC than in mice treated with 8 mg/L NTBC (*p* = 0.035), while tryptophan concentrations were not statistically different. Comparisons between FAH−/− mice treated with 8 mg/L or 32 mg/L NTBC to the mice receiving the same amount of NTBC and diet, revealed that brain phenylalanine concentrations were within the same range, while brain tyrosine concentrations were lower (*p* < 0.001) and brain tryptophan concentrations were not different. In both groups of dietary treated FAH−/− mice, brain phenylalanine concentrations were lower than concentrations found in untreated WT mice (*p* < 0.01), while brain tyrosine concentrations were still significantly higher (*p* < 0.001). When WT mice treated with NTBC were compared to untreated WT mice, brain phenylalanine and tryptophan were not different, while tyrosine concentrations were substantially higher (*p* < 0.001). Figure 3 shows all other brain LNAA concentrations. In general, brain LNAA concentrations tended to be lower in FAH−/− mice treated with NTBC alone when compared to WT mice, especially with regard to brain valine, isoleucine and threonine concentrations. Except for brain histidine and threonine concentrations, brain LNAA concentrations were not significantly different between dietary treated FAH−/− mice and WT mice.

**Figure 4:**
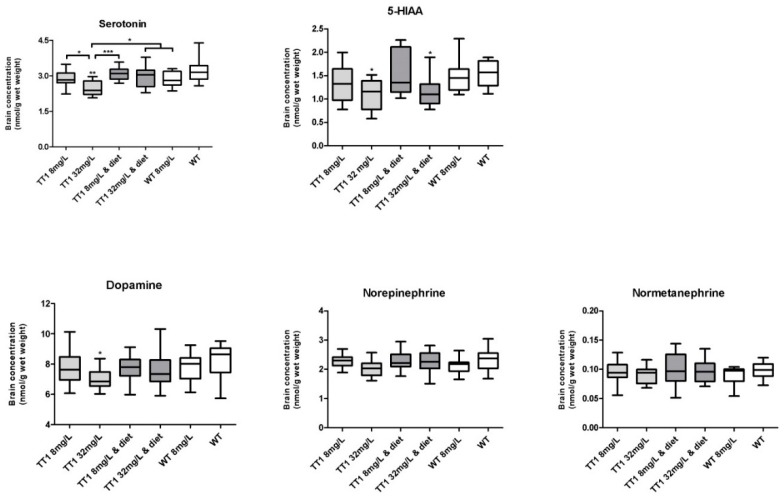
Concentrations of brain neurotransmitters and its metabolites. Numbers of mice are *n* = 13 or *n* = 14 for all treatment groups. Untransformed data are expressed as boxplots (min–max whiskers). * *p* < 0.05, ** *p* < 0.01 and *** *p* < 0.001 (two-sided).

Figure 4 shows brain neurotransmitter concentrations in WT and FAH−/− mice treated by different doses of NTBC with or without diet. FAH−/− mice treated with 32 mg/L NTBC had significantly lower brain serotonin concentrations when compared to all other group of mice (*p* < 0.05) and significantly lower brain 5-hydroxyindoleacetic acid (5-HIAA) concentrations when compared to untreated WT mice (*p* = 0.019). FAH−/− mice treated with 32 mg/L NTBC and diet had lower brain 5-HIAA concentrations when compared to untreated WT mice (*p* = 0.015). Brain dopamine concentrations were significantly lower in FAH−/− mice treated with 32 mg/L when compared to untreated WT mice (*p* = 0.018). No significant difference in serotonin and dopamine concentrations between the other groups of mice could be found. In addition to this, norepinephrine and normetanephrine concentrations were not significantly different between the different groups of mice.

### 3.4. Association between Different Biochemical Parameters

**Figure A1:**
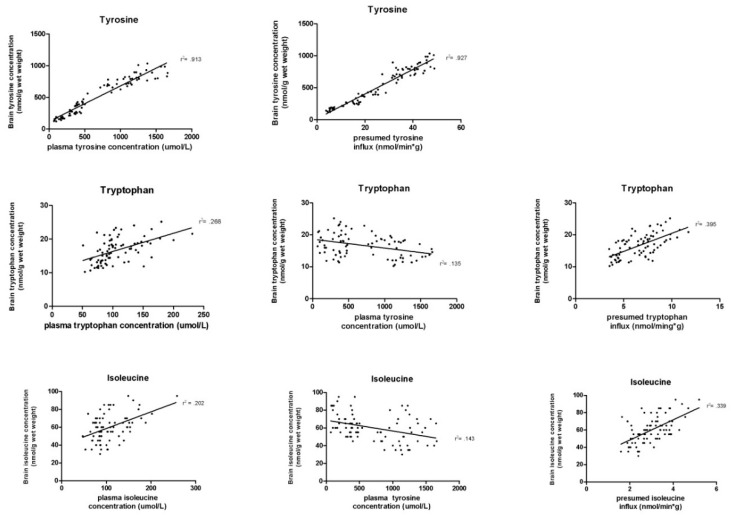
Correlations between several brain LNAA and its own plasma concentration, plasma tyrosine concentration and the calculated presumed brain influx. The different graphs show that most of the variance (displayed as *R*^2^) is explained when the presumed brain influx is correlated to the brain LNAA concentration.

All individual brain LNAA concentrations were positively associated with its plasma concentration (median: *p* < 0.001, *F =* 22.0; range: *p* < 0.001–*p* = 0.001; *F* = 838.7–11.4) and most of the non-tyrosine brain LNAA (except methionine and histidine) were negatively associated with plasma tyrosine concentrations (median: *p* = 0.006, *F* = 9.6.; range: *p* < 0.001–*p* = 0.677; *F* = 47.4–0.2). In addition to this, all brain LNAA concentrations were positively associated with the calculated presumed brain influx. Most brain LNAA (except methionine and histidine) were most strongly associated with the presumed brain influx (median: *p* < 0.001, *F* = 38.4; range: *p* < 0.001–*p* = 0.002; *F* = 1010.0–10.1) and not to its own plasma or plasma tyrosine concentrations (Appendix A, Figure A1).

Brain tryptophan concentrations were not significantly associated to brain serotonin concentrations (*p* = 0.559; *F* = 0.344). Brain tyrosine concentrations were significantly negatively associated with brain dopamine (*p =* 0.016; *F* = 5.702), brain norepinephrine (*p* = 0.007; *F* = 7.563) and brain normetanephrine (*p* = 0.048; *F* = 4.053) and negatively associated with brain serotonin concentrations (*p* < 0.001; *F* = 18.962).

### 3.5. Behavioral Tests

**Figure 5:**
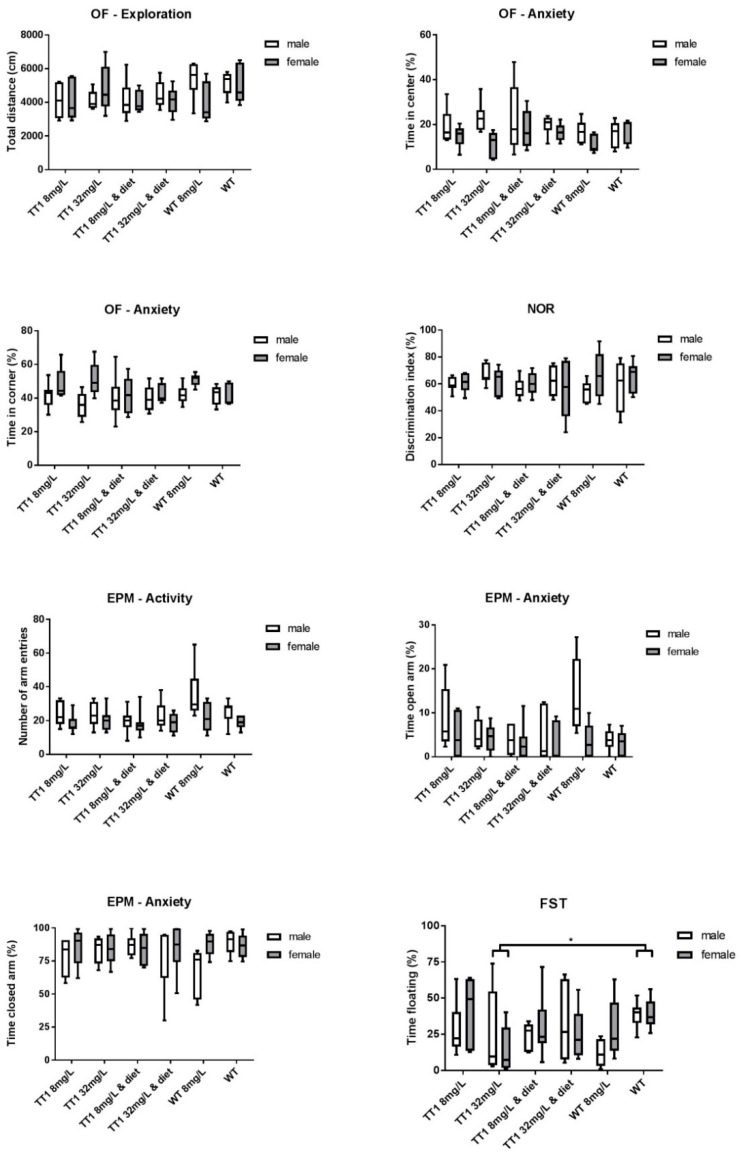
Behavioral outcomes on the open field (OF), novel object recognition (NOR), elevated plus maze (EPM) and forced swim test (FST) comparing the different treatment groups. Although all mice underwent behavioral testing, the number of mice vary between *n* = 10 and *n* = 14 for all treatment groups in the different tests. Untransformed data are expressed as boxplots (min–max whiskers). * *p* < 0.05 (two-sided).

Figure 5 shows results on the different behavioral tests in WT and FAH−/− mice treated by different doses of NTBC with or without diet. The total distance moved during the OF test did not significantly differ between the different groups of mice (*p* = 0.201). The time spent in the center zone and corners of the field did not differ between the different group of mice (*p* = 0.423 and *p* = 0.496 respectively), although females were more anxious and spent more time in the corners and less in the center of the OF when compared to male mice (*p* = 0.004 and *p* = 0.007). In the NOR, the mean discrimination index was above 50% in all groups of mice and did not significantly differ between the different groups of mice (*p =* 0.931). In the EPM, females had significantly less total entries (*p* = 0.002) and in accordance with results on the OF test, females spent less time in the open arms compared to males (*p* = 0.018), but no significant differences between the different treatment groups were found. The percentage of time spent floating during the FST was significantly higher in untreated WT mice compared FAH−/− mice treated with 32 mg/L NTBC (*p* = 0.034).

## 4. Discussion

This is the first study investigating (1) plasma and brain biochemistry in NTBC treated FAH−/− mice, (2) evaluating the effect of different doses of NTBC and dietary treatment on plasma and brain biochemistry and (3) assessing the behavioral outcome of mice following the different treatment regimes. The main findings of this study are six-fold. Firstly, NTBC treated FAH−/− mice show high plasma tyrosine and low plasma phenylalanine concentrations. Secondly, these changes in plasma LNAA concentrations led to high brain tyrosine concentrations while lower brain concentrations of some other LNAA could be found. Brain LNAA concentrations did not only depend on its own plasma concentration, but most of all on competitive exchange of LNAA at the blood brain barrier. Thirdly, neurotransmitter concentrations in FAH−/− were mostly normal, although especially serotonin concentrations were negatively correlated with brain tyrosine concentrations. Fourthly, a phenylalanine-tyrosine restricted diet could lower plasma and brain tyrosine concentrations and normalize most brain LNAA and neurotransmitter concentrations. Fifthly, quantitatively detectable blood SA concentrations could not only be prevented by increasing the dose of NTBC but also by dietary restriction of phenylalanine and tyrosine. Furthermore, and lastly, despite clear cognitive deficiencies in TT1 patients, no clear behavioral problems were found in these FAH−/− mice on NTBC with or without a diet.

Treatment with NTBC clearly increased plasma tyrosine concentrations in all mice, while phenylalanine concentrations tended to decrease. This lowering effect of NTBC on plasma phenylalanine concentrations has been found in other studies as well, although the pathophysiological mechanism is not clear yet [18]. Dietary restriction lowered plasma tyrosine concentrations, without completely normalizing it, while plasma phenylalanine concentrations tended to decrease even further. This is in accordance with plasma tyrosine and phenylalanine concentrations in TT1 patients, although phenylalanine concentrations in this study were not as low as has been described in TT1 infants in which they were causing growth and developmental problems in infancy [19]. In contrast to the amino acid mixtures used in TT1 patients, some phenylalanine was added to the diet of the mice to prevent growth problems caused by extremely low phenylalanine concentrations. In this way, these extremely low phenylalanine concentrations could be prevented in the dietary treated FAH−/− mice. However, as a consequence, possible behavioral problems caused by low phenylalanine concentrations were difficult to examine. Some other plasma LNAA tended to be decreased in FAH−/− animals especially when treated with NTBC only. This could possibly be attributed to competition by tyrosine at the gut-blood barrier in a similar way as has been seen with phenylalanine in PKU mice [20].

In TT1 patients, brain LNAA and neurotransmitters concentrations have been estimated based on cerebral spinal fluid (CSF) measurements [6] or theoretical calculations [7]. In agreement with these estimations, this study clearly shows that high plasma tyrosine concentrations led to high brain tyrosine concentrations. Dietary treatment clearly reduces the brain tyrosine concentrations, although normal concentrations are never reached. This could be of importance as high brain tyrosine concentrations have been considered neurotoxic, causing oxidative stress, deoxyribonucleic acid damage and changes in mitochondrial energy metabolism in several rat brain regions [21,22,23]. However, the high brain tyrosine concentrations did not induce behavioral problems in these FAH−/− and WT mice.

In addition to high brain tyrosine concentrations, the competitive effect of high plasma tyrosine concentrations at the blood–brain barrier in combination with the slightly decreased plasma concentrations of the other non-tyrosine LNAA led to low non-tyrosine brain LNAA concentrations. This was especially true for brain phenylalanine, valine and isoleucine concentrations, where FAH−/− mice treated with NTBC alone showed the lowest concentrations. Dietary treatment normalized all brain LNAA concentrations, except for brain phenylalanine concentrations which were clearly decreased. Important to notice is that despite the increased brain tyrosine concentrations, most non-tyrosine brain LNAA concentrations were usually only moderately affected in the different groups of FAH−/− mice, especially when compared to the clearly decreased brain LNAA in PKU and MSUD mice [8,9]. This could be explained by the kinetics of the LAT-1 transporter, as tyrosine has a lower affinity for the LAT-1 transporter than phenylalanine and leucine. Changes in tyrosine concentrations are therefore less detrimental for brain LNAA concentrations than changes in phenylalanine or leucine concentrations [16,24]. Still, the importance of the competitive effect of the blood brain barrier in determining the brain LNAA concentrations is shown as all brain LNAA were most of all correlated with the calculated theoretical brain influx and not solely with its own plasma concentrations.

As tyrosine is the precursor for the catecholaminergic neurotransmitters dopamine and norepinephrine, high brain tyrosine concentrations were thought to change neurotransmitter concentrations and/or concentrations of its metabolites [4]. However, despite high brain tyrosine concentrations, catecholaminergic neurotransmitter concentrations were normal (or even slightly lower) in FAH−/− mice. This could be explained as the activity of tyrosine hydroxylase, the rate limiting enzyme for catecholaminergic neurotransmitter synthesis, is tightly regulated, among others by substrate and product inhibition allowing dopamine concentrations to stay within range [25,26,27,28]. Regarding indolamines, although brain tryptophan concentrations did not differ between the different groups of mice, serotonin and 5-HIAA were reduced in some of the FAH−/− mice and turned out to be negatively correlated with brain tyrosine concentrations. At least in one study, brain tyrosine (and dopamine) has been shown not only to inhibit tyrosine hydroxylase but tryptophan hydroxylase, the rate limiting enzyme in serotonin synthesis, as well [29]. In this way, FAH−/− mice could be prone to develop a serotonin deficiency when experiencing high brain tyrosine concentrations. Thimm et al. showed similar results in CSF of TT1 patients, measuring normal concentrations of dopamine metabolite homovanillic acid and low concentrations of 5-HIAA suggesting a serotonin deficiency in TT1 patients [6].

As described earlier, FAH−/− mice need a higher daily dose of NTBC than patients to prevent increased SA concentrations [30]. The general recommendation is to treat patients with 1 (to 2) mg/kg/day NTBC [1,2], which would be comparable to the amount received when mice drink 8 mg/L NTBC drinking water. In this study, quantitatively detectable SA concentrations were often found with this dose of NTBC, which is in accordance to earlier studies and could most likely be attributed to the higher metabolic rate, differences in biological bioavailability, or other differences in the tyrosine catabolic pathway in mice when compared to humans [30]. However, this study also shows that quantitatively detectable SA concentrations in the FAH−/− mice could not only be prevented by increasing the NTBC dose, but also by reducing the flux through the phenylalanine-tyrosine catabolic pathway by a phenylalanine and tyrosine restricted diet. These findings highlight the competitive inhibition of HPPD by NTBC. Taken all species differences into account, this finding could stress the importance of close monitoring of the adequacy of NTBC treatment in TT1 patients, especially when adherence to the diet is non-optimal.

Although NTBC treated TT1 patients showed various cognitive problems [10,31,32,33,34,35], these FAH−/− mice showed no clear behavioral deficits despite the biochemical differences between the different groups of mice. With the different experimental groups, we tried to reveal behavioral deficits possibly caused by (1) the disease itself and associated metabolites that are at least known to be hepatotoxic, (2) high brain tyrosine concentrations that are possibly toxic or outcompete the brain uptake of other LNAA, (3) low plasma and brain phenylalanine concentrations and (4) NTBC (mice treated with high dose NTBC). As FAH−/− mice required prenatal NTBC administration, untreated WT mice had to be obtained by a separate breeding instead of using littermates. However, to prevent behavioral changes, all mice were housed in the same room and handled by the same person. Previous research, conducted with a different strain of mice, showed that FAH−/− mice had social problems that were associated with the disease itself and not with its treatment [12]. Although these mice showed a slower learning curve, their memory retention was unaffected [11]. This is in agreement with our study showing that explorative behavior, anxiety and memory retention were unaffected in FAH−/− mice treated with NTBC and diet.

## 5. Conclusions

To conclude, increased plasma tyrosine concentrations in FAH−/− mice lead to high brain tyrosine concentrations. The phenylalanine and tyrosine restricted diet seems to be important not only to lower plasma and resulting brain tyrosine concentrations, but also to prevent small changes in brain LNAA and serotonin concentrations and maybe even to prevent possible detectable SA in blood. Further research in FAH−/− mice should study if a stricter diet would induce possible side effects such as deficiencies in phenylalanine that could not be observed in the present study, and may result in the neurocognitive profile as seen in patients [10,19].

## Abbreviations

NTBC	2-(2-Nitro-4-trifluoromethylbenoyl)-1,3-cyclohexanedione
HPPD	4-hydroxyphenylpyruvate dioxygenase
5-HIAA	5-hydroxyindoleacetic acid
EPM	elevated plus maze
FST	forced swim test
FAH	fumarylacetoacetate hydrolase
LNAA	large neutral amino acids
MSUD	maple syrup urine disease
NOR	novel object recognition
OF	open field
PKU	phenylketonuria
TT1	tyrosinemia type 1
SA	succinylacetone
WT	wild-type
